# Potential effects of adverse childhood experiences on school engagement in youth: a dominance analysis

**DOI:** 10.1186/s12889-022-14524-8

**Published:** 2022-11-16

**Authors:** Nathaniel J. Webb, Thaddeus L. Miller, Erica L. Stockbridge

**Affiliations:** 1 Department of Health Behavior and Health Systems, School of Public Health, University of North Texas Health Science Center, 3500 Camp Bowie Blvd, Fort Worth, TX 76107 USA; 2 Department of Internal Medicine, Texas College of Osteopathic Medicine, University of North Texas Health Science Center, 3500 Camp Bowie Blvd, Fort Worth, TX 76107 USA

**Keywords:** Adverse childhood experiences, School engagement, Dominance analysis, Parental incarceration, Economic hardship

## Abstract

**Background:**

Adverse childhood experiences (ACEs) can have severe negative impacts on childhood and adult health via worsened school engagement and educational outcomes. This study seeks to identify the relative importance of various ACEs in predicting school engagement.

**Methods:**

We analyzed data from the National Survey of Children’s Health for school-aged children (ages 6-17) for 2018 and 2019. The primary outcome was school engagement, measured through three variables: repeating a grade, doing required homework, and caring about doing well in school. We conducted three logistic regression models with dominance analyses to identify the relative importance of ACE variables in predicting school engagement outcomes.

**Results:**

In unadjusted and adjusted dominance analyses, parental incarceration was the most important ACE in predicting repeating a grade. Living in a household in which it was hard to cover basics like food or housing was the most important ACE in predicting doing required homework and caring about doing well in school.

**Discussion:**

Our study points toward the large influence of out-of-school factors on school engagement. Parental incarceration and economic hardship, the most important predictors of engagement, are issues that can be addressed and mitigated through policy interventions. With limited funds available for education and public health interventions, it is crucial that these two ACEs be priority considerations when developing policy. A multi-faceted approach that reduces the incarcerated population, encourages economic well-being, and emphasizes early-childhood education has the potential to significantly improve school engagement in vulnerable populations and ultimately advance social equity.

**Supplementary Information:**

The online version contains supplementary material available at 10.1186/s12889-022-14524-8.

## Background

Preventing and mitigating the effects of Adverse Childhood Experiences (ACEs) is a priority for the US Centers for Disease Control and Prevention (CDC) because ACEs can have striking negative impacts on health [1]. ACEs are potentially traumatic events that occur before age 17, such as experiencing or witnessing violence in the family or community [2]. ACEs and early childhood stress influence developing bodies and brains of adolescents, and can result in short- and long-term negative consequences such as learning, behavioral, and physiological issues [3–5]. ACEs are associated with altered stress responses where individuals are more sensitive to stress later in life [6]. This stress sensitivity results in individuals being particularly vulnerable to stress-related diseases including mental and somatic conditions [6]. Consequently, ACEs are associated with worse health during all stages of life [7–12]. Further, ACEs are directly and indirectly associated with worse school engagement and educational attainment during adolescence [7–12].. Educational attainment is a fundamental and well-described determinant of health [13], and ACEs effect on education can compound their lasting negative impact. ACEs can influence school engagement, and increasing numbers of ACEs are associated with decreasing levels of school engagement [7, 11, 12]. While the total number of ACEs and specific ACEs have been investigated, little evidence exists to evaluate the level of influence each contributes to school engagement.

As funding for school engagement interventions is limited, it is important to identify the most influential ACEs on predicting school engagement to maximize the benefits of future programs. Additionally, each ACE can be influenced to varying degrees through interventions, so identifying the most important ACE variables will help determine the best way to address the school engagement issue. While there is a vast body of research on ACEs, none has examined the relative importance of ACEs on an outcome using dominance analysis, and we evaluated and ranked selected ACEs’ influence in predicting school engagement with that technique. Existing research has used regression analyses to identify different ACEs and number of ACEs as predictors of school engagement; this study seeks to contextualize previous research by identifying the ranked order of importance of ACE variables on predicting school engagement. Findings will provide evidence to prioritize preventive interventions, inform public health policy decisions, and guide future research. Parental incarceration and economic hardship have been the primary focus of previous research, and thus, we hypothesize that these two ACEs will be most influential in predicting school engagement [14, 15].

## Methods

### Dataset

Data came from the National Survey of Children’s Health (NSCH) for 2018 and 2019. NSCH is a national survey conducted by the US Census Bureau and sponsored by the US Department of Health and Human Services’ Health Resources and Services Administration Maternal and Child Health Bureau. The survey investigated the health and well-being of non-institutionalized children ages 0-17 and was administered in web-based and paper format [16]. Households were randomly sampled using a complex sampling design, with parental or guardian surrogates completing the survey for one child in the household for all of the included variables [16]. In 2018 and 2019, 59,963 surveys were completed [16]. We used guidance provided by the US Census Bureau to combine, adjust, and weight the two years of data to yield nationally representative estimates [17].

Because of our focus on school engagement, analyses were restricted to children ages 6-17 (*N* = 43,213), with a mean unadjusted age of 12.11 years. Children with missing data on any of the variables discussed below were excluded from analysis, with the final dataset containing 39,347 individuals (52.1% male and 47.9% female). Additionally, the race and ethnicity distribution were 70.4% non-Hispanic White, 11.5% Hispanic, 6.2% non-Hispanic Black, 6.0% non-Hispanic other, and 6.0% two or more races. This project was reviewed and approved as exempt category human subjects research by the North Texas Regional Institutional Review Board.

### Outcome variables

We examined three measures of school engagement. The first outcome variable we investigated was repeating a grade in school, which was measured with the following question: “Since starting kindergarten, has this child repeated any grades?” Responses included yes/no. The second outcome variable examined children’s interest in doing well in school: “How often does this child care about doing well in school?” Our final outcome variable explored whether children completed all required homework: “How often does the child do all required homework?” The responses to the second and third outcome variables were always/usually/sometimes/never; we dichotomized these responses in accordance with past research [12, 18].

### Main explanatory variables

Nine different ACEs were assessed for the NSCH. They were economic hardship, parental/guardian incarceration, parental/guardian death, parental/guardian divorce, being a victim of or witnessing violence in the neighborhood, witnessing adult violence in the home, living with someone who was mentally ill, living with someone with a substance abuse issue, and discrimination on the basis of race or ethnicity. Detailed descriptions of these ACEs and how they were ascertained are available elsewhere (https://www.census.gov/programs-surveys/nsch.html).

### Explanatory covariates

We included a number of covariates in our analyses to control for potential confounders. These included the demographic variables sex, age, race, and ethnicity. Race is a social construct that interacts with the educational system [19]. Racial inequities influence educational outcomes [19]. Consequently, race and ethnicity are included throughout adjusted analyses. Additional covariates include the lowest household parental educational attainment and children with special health care needs (SHCN).

### Statistical analysis

We produced descriptive statistics on the variables mentioned above and used chi-square analyses to identify unadjusted associations between our explanatory variables and outcomes. We estimated the adjusted associations between the explanatory variables and each outcome in three multiple logistic regression models. These models were subsequently used in dominance analyses to identify the relative importance of the main explanatory variables on predicting the school engagement outcome variables. We tested the multicollinearity assumption of logistic regression to identify any potential collinearity of the explanatory and covariables (Additional file 1).

Dominance analysis is a statistical method that ranks the importance of each of the main explanatory variables in predicting an outcome. This analysis considers the explanatory variable’s unique effect on the outcome as well as its joint effect with other variables [20]. Importance is determined by running a series of regression models (termed “submodels”), which incorporate each potential combination of explanatory variables and averages the change in the McFadden pseudo-R^2^ values for each combination [20–22]. This average is termed a “general dominance statistic,” and reflects predictive strength; explanatory variables with a larger general dominance statistic are of greater importance than those with a relatively smaller general dominance statistic [20].

Three dominance analyses were conducted: one for each outcome variable. Covariates were held constant across all dominance analyses; only the importance of the ACE variables was examined. A model with *p* predictors requires the calculation of 2^*p*^-1 submodels to estimate dominance [22], resulting in 511 different submodels for each dominance analysis. All data analysis was conducted using Stata SE version 15.1 (College Station, TX). We accounted for complex survey design and set the significance level at *p* < 0.05.

## Results

### Demographics

**Fig. 1:**
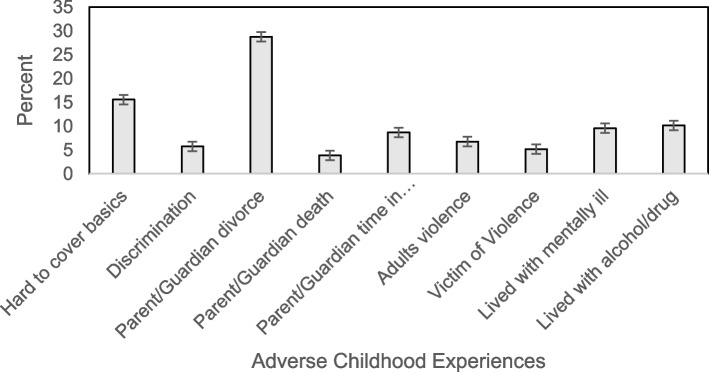
Prevalence of reported Adverse Childhood Experiences from the National Survey of Children’s Health (2018-2019)

Our sample (*n* = 39,347) was weighted to be nationally representative of the US population for children ages 6-17. The most prevalent ACE was parental or guardian divorce at 28.8% (95% CI: 27.8, 29.8) of the population. This was followed by hard to cover basics like food or housing (15.6, 95% CI: 14.8, 16.5), living with someone with a substance abuse issue (10.2, 95% CI: 9.6, 10.9), living with someone with a mental illness (9.6, 95% CI: 9.0, 10.3), parental incarceration (8.7, 95% CI: 8.1, 9.3), witnessing adult violence (6.8, 95% CI: 6.2, 7.3), treated unfairly because of race (5.8, 95% CI: 5.3, 6.4), and victim of violence (5.2, 95% CI: 4.7, 5.7). The least prevalent ACE was parental or guardian death at 3.9% (95% CI: 3.5, 4.3) of the population (Fig. 1). Descriptive statistics for demographic, covariate, and ACE variables are detailed in Additional file 2.

Overall, 6.3% (95% CI: 5.7, 6.9) of the population repeated a grade at some point during their education. Additionally, 88.7% (95% CI: 88.0, 89.3) of the population always/usually did all required homework while 11.3% (95% CI: 10.7, 12.0) sometimes/never did all required homework. Finally, 89.1% (95% CI: 88.5, 89.7) of the population always/usually cared about doing well in school while 10.9% (95% CI: 10.3, 11.5) sometimes/never cared about doing well in school. Chi-squared analyses found significant differences between groups for all three outcomes for all demographic, covariate, and ACE variables (*p* < 0.001, Additional file 2).

### Logistic regression

#### Model 1: repeated grades

In the first logistic regression model, we found a significant association between sex, age group, race/ethnicity, health issues, minimum parental education, ACE variables, and the repeated grades outcome. Of the nine ACE variables, three were significant: participants that experienced challenges in meeting basic needs (OR: 1.3, 95% CI: 1.0, 1.6), parental or guardian death (OR: 1.7, 95% CI: 1.1, 2.7), and parental or guardian incarceration (OR: 1.5, 95% CI: 1.1, 2.1) had increased odds of repeating a grade. Table 1 details all logistic regression results for Model 1.

**Table 1:** Adjusted odds ratios of sociodemographic covariates and ACE predictors of repeated grade, does all required homework, and cares about doing well in school

Variable	Model 1: Repeated Grade			Model 2: Does All Required Homework			Model 3: Cares About Doing Well in School		
	Odds Ratio	95% Confidence Interval	*P*-value	Odds Ratio	95% Confidence Interval	P-value	Odds Ratio	95% Confidence Interval	P-value
Demographic Covariates									
Sex									
Male	1.00 (Ref)			1.00 (Ref)			1.00 (Ref)		
Female	0.6	(0.5, 0.7)	< 0.0001	2.0	(1.7, 2.3)	< 0.0001	2.1	(1.8, 2.4)	< 0.0001
Age									
6-9	1.00 (Ref)			1.00 (Ref)			1.00 (Ref)		
10-13	1.6	(1.2, 2.1)	0.001	0.7	(0.6, 0.9)	0.005	1.0	(0.8, 1.1)	0.634
14-17	2.2	(1.7, 2.8)	< 0.0001	0.5	(0.4, 0.7)	< 0.0001	0.8	(0.7, 0.9)	0.009
Race/Ethnicity									
NH White	1.00 (Ref)			1.00 (Ref)			1.00 (Ref)		
NH Black	1.4	(1.1, 1.8)	0.009	0.7	(0.5, 0.8)	< 0.0001	0.9	(0.7, 1.1)	0.416
Hispanic	1.2	(0.9, 1.6)	0.255	1.0	(0.8, 1.2)	0.719	1.1	(0.9, 1.3)	0.547
NH Other	1.0	(0.7, 1.5)	0.876	0.9	(0.7, 1.2)	0.632	0.9	(0.7, 1.1)	0.327
> =2 Races, NH	1.0	(0.7, 1.5)	0.938	1.4	(1.0, 1.9)	0.058	1.1	(0.8, 1.5)	0.701
Health Issues									
None	1.00 (Ref)			1.00 (Ref)			1.00 (Ref)		
Asthma, no ADHD	1.0	(0.7, 1.3)	0.839	0.7	(0.5, 0.9)	0.004	0.6	(0.5, 0.8)	0.001
ADHD, no asthma	3.1	(2.4, 4.0)	< 0.0001	0.2	(0.1, 0.2)	< 0.0001	0.1	(0.1, 0.2)	< 0.0001
Both Asthma, ADHD	2.6	(1.6, 4.0)	< 0.0001	0.2	(0.1, 0.2)	< 0.0001	0.2	(0.1, 0.3)	< 0.0001
Neither, other SHCN	2.2	(1.7, 2.9)	< 0.0001	0.3	(0.3, 0.4)	< 0.0001	0.3	(0.2, 0.4)	< 0.0001
Minimum Parent Education									
Less than HS	1.00 (Ref)			1.00 (Ref)			1.00 (Ref)		
HS/GED/Voc	0.6	(0.5, 0.8)	0.002	1.2	(1.0, 1.5)	0.121	1.1	(0.9, 1.4)	0.39
Some college	0.4	(0.3, 0.5)	< 0.0001	1.4	(1.1, 1.8)	0.008	1.3	(1.0, 1.7)	0.022
Bachelors	0.3	(0.2, 0.4)	< 0.0001	1.6	(1.2, 2.1)	0.001	1.7	(1.3, 2.2)	< 0.0001
Masters or more	0.2	(0.1, 0.3)	< 0.0001	2.1	(1.4, 2.9)	< 0.0001	2.3	(1.7, 3.1)	< 0.0001
Adverse Childhood Experiences									
Basic Needs									
Yes	1.3	(1.0, 1.6)	0.039	0.5	(0.4, 0.6)	< 0.0001	0.6	(0.5, 0.7)	< 0.0001
Racism									
Yes	1.4	(1.0, 2.1)	0.054	0.7	(0.5, 1.0)	0.023	0.9	(0.7, 1.2)	0.477
Divorce									
Yes	1.1	(0.8, 1.3)	0.636	0.8	(0.6, 0.9)	< 0.0001	0.7	(0.6, 0.8)	< 0.0001
Parental Death									
Yes	1.7	(1.1, 2.7)	0.017	1.0	(0.7, 1.3)	0.8	0.9	(0.7, 1.2)	0.523
Parental Incarceration									
Yes	1.5	(1.1, 2.1)	0.004	0.8	(0.6, 1.0)	0.046	0.9	(0.7, 1.1)	0.274
Adult Violence									
Yes	1.3	(0.9, 1.8)	0.15	0.9	(0.7, 1.2)	0.564	1.1	(0.8, 1.4)	0.628
Neighborhood Violence									
Yes	0.8	(0.6, 1.2)	0.366	0.8	(0.6, 1.0)	0.068	0.7	(0.6, 1.0)	0.037
Lived with Mental Health									
Yes	1.3	(1.0, 1.8)	0.098	0.8	(0.6, 1.0)	0.015	0.8	(0.7, 1.0)	0.107
Lived with Substance Abuse									
Yes	0.9	(0.6, 1.5)	0.813	1.0	(0.8, 1.3)	0.988	0.9	(0.7, 1.1)	0.227

#### Model 2: does all required homework

In the second logistic regression model, we found a significant association between sex, age group, race/ethnicity, health issues, minimum parental education, ACE variables, and the does all required homework outcome. Children in households that experienced challenges in meeting basic needs (OR: 0.5, 95% CI: 0.4, 0.6), racism (OR: 0.7, 95% CI: 0.5, 1.0), parental divorce (OR: 0.8, 95% CI: 0.6, 0.9), parental or guardian incarceration (OR: 0.8, 95% CI: 0.6, 1.0), or having lived with someone with a mental illness (OR: 0.8 95% CI: 0.6, 1.0) had significantly lower odds of doing required homework. Table 1 details all logistic regression results for Model 2.

#### Model 3: cares about doing well in school

In the third logistic regression model, we found a significant association between sex, age group, health issues, minimum parental education, ACE variables, and the cares about doing well in school outcome. Children in households that experienced challenges in meeting basic needs (OR: 0.6, 95% CI: 0.5, 0.7), parental divorce (OR: 0.7, 95% CI: 0.6, 0.8), or having been a victim of violence (OR: 0.7, 95% CI: 0.6, 1.0) had significantly lower odds of caring about doing well in school. Table 1 details all logistic regression results for Model 3.

### Dominance analysis

#### Model 1: repeated grades

In both unadjusted and adjusted dominance analysis, parental incarceration was the most dominant ACE for predicting repeating a grade, accounting for 22.6% (unadjusted) or 3.1% (adjusted) of the change in pseudo-R^2^ values. In unadjusted analyses, hard to cover basic needs like food or housing (2) and parental death (3) were the next most dominant variables in predicting repeating a grade. These two variables were also important in adjusted analyses but the order shifted, with parental death (2) and hard to cover basic needs like food or housing (3) being the second and third most dominant variables. Table 2 include all dominance analysis results for Model 1.

**Table 2:** Unadjusted and adjusted dominance analyses for repeated grade logistic regression model. Unadjusted model does not control for demographic, parental education, or health issues

Variable	Unadjusted			Adjusted		
	Dominance Statistic	Standardized Dominance Statistic	Ranking	Dominance Statistic	Standardized Dominance Statistic	Ranking
Close with an individual with substance use issue	0.002	0.046	8	0.001	0.005	8
Divorce	0.003	0.090	6	0.001	0.006	7
Experienced racism/discrimination	0.003	0.089	7	0.002	0.014	6
Hard to cover basics like food or housing	0.005	0.153	2	0.002	0.016	3
Lived with someone with a mental illness	0.004	0.108	5	0.002	0.015	5
Parental Death	0.004	0.130	3	0.003	0.023	2
Parental Incarceration	0.007	0.226	1	0.003	0.031	1
Victim of violence	0.001	0.045	9	0.000	0.002	9
Witnessed adult violence	0.004	0.114	4	0.002	0.015	4

#### Model 2: does all required homework

In both unadjusted and adjusted dominance analysis, covering basic needs was the most dominant ACE for predicting doing all required homework, accounting for 28.4% (unadjusted) or 6.4% (adjusted) of the change in pseudo-R^2^ values. In both unadjusted and adjusted analyses, parental divorce (2) and lived with someone with a mental illness (3) were the next most dominant variables in predicting doing all required homework. Table 3 include all dominance analysis results for Model 2.

**Table 3:** Unadjusted and adjusted dominance analyses for does all required homework logistic regression model. Unadjusted model does not control for demographic, parental education, or health issues

Variable	Unadjusted			Adjusted		
	Dominance Statistic	Standardized Dominance Statistic	Ranking	Dominance Statistic	Standardized Dominance Statistic	Ranking
Close with an individual with substance use issue	0.003	0.050	8	0.001	0.007	8
Divorce	0.010	0.165	2	0.004	0.023	2
Experienced racism/discrimination	0.004	0.065	7	0.002	0.012	6
Hard to cover basics like food or housing	0.016	0.284	1	0.010	0.064	1
Lived with someone with a mental illness	0.008	0.143	3	0.003	0.016	3
Parental Death	0.001	0.011	9	0.000	0.001	9
Parental Incarceration	0.006	0.105	5	0.002	0.014	5
Victim of violence	0.006	0.108	4	0.002	0.014	4
Witnessed adult violence	0.004	0.070	6	0.001	0.009	7

#### Model 3: cares about doing well in school

In both unadjusted and adjusted dominance analysis, covering basic needs was the most dominant ACE for predicting caring about doing well in school, accounting for 29.5% (unadjusted) or 5.2% (adjusted) of the change in pseudo-R^2^ values. In both unadjusted and adjusted analyses, parental divorce (2) was the next most dominant variable in predicting caring about doing well in school. The third most dominant variable shifted between unadjusted (lived with someone with a mental illness) and adjusted (victim of violence) analyses. Table 4 include all dominance analysis results for Model 3.

**Table 4:** Unadjusted and adjusted dominance analyses for cares about doing well in school logistic regression model. Unadjusted model does not control for demographic, parental education, or health issues

Variable	Unadjusted			Adjusted		
	Dominance Statistic	Standardized Dominance Statistic	Ranking	Dominance Statistic	Standardized Dominance Statistic	Ranking
Close with an individual with substance use issue	0.004	0.079	6	0.002	0.011	4
Divorce	0.009	0.194	2	0.004	0.027	2
Experienced racism/discrimination	0.001	0.021	8	0.001	0.003	8
Hard to cover basics like food or housing	0.014	0.295	1	0.008	0.052	1
Lived with someone with a mental illness	0.007	0.144	3	0.002	0.010	5
Parental Death	0.001	0.012	9	0.000	0.001	9
Parental Incarceration	0.004	0.090	5	0.001	0.008	6
Victim of violence	0.005	0.108	4	0.002	0.013	3
Witnessed adult violence	0.003	0.057	7	0.001	0.004	7

## Discussion

We identified the relative importance of ACE variables on predicting school engagement in school-aged children as measured by repeating a grade, doing all required homework, and caring about doing well in school. We found that parental incarceration was the most dominant variable for predicting repeating a grade, while difficulty covering basic needs like food or housing was the most dominant for predicting doing all required homework and caring about doing well in school. Consequently, children with incarcerated parents and those in households without the ability to meet basic needs should be prioritized when developing interventions to improve school engagement. Additionally, strategies that decrease the likelihood of parental incarceration and increase the ability of households with children to meet basic needs are most likely to improve school engagement.

Our findings regarding the importance of parental incarceration in predicting repeating a grade align with previous research showing the negative effects of mass incarceration in the US on education and academic outcomes for children of those incarcerated [14, 23, 24]. The association between school engagement and parental incarceration is of concern given societal trends in incarceration. Since 1980, the incarcerated population in the US has grown exponentially, resulting in the highest incarceration rate in the world [25]. Mass incarceration is a structural inequity disproportionately impacting low-income communities and communities of color [26, 27]. Consequently, children in low-income households and children of color will be disproportionately affected by the negative educational outcomes associated with parental incarceration.

Children in low-income households also risk lower educational engagement as measured by other outcomes, and our analysis illuminates the influence of economic hardship on school engagement. We found that having had trouble meeting basic needs like food or housing played an important role in each measured school engagement outcome, and economic hardship was the most dominant variable in predicting whether children do all required homework or care about doing well in school. Previous research identified the importance Socio-Economic Status (SES) plays in school engagement and educational attainment [28–30]. Children with higher SES have shown higher levels of school engagement and thus graduation rates [28]. Additionally, previous research identified the impact food insecurity has on health status, emotional well-being, and school engagement [29, 30].

There is an association between economic pressure and parental conflict [30], and this can lead to parental divorce. Further, children living in a single-parent household are more likely to live in low-income households [31]. Given the associations between economic hardship and other aspects of a family’s life, it is not surprising that this variable emerged as dominant in our analyses. Economic hardship influences or is influenced by many of the other ACEs in our analysis. Our results indicate that, while economic hardship is generally a stronger predictor, family structure ACEs are also important predictors of children repeating a grade and doing all required homework. Our study provides new evidence regarding the link between economic hardship, family structure, and school engagement. Previous research shows the negative associations between parental death and school performance [32] and between parental divorce and lower educational attainment [33].

Policy changes are needed at the state and federal levels for a successful reduction in the incarcerated population. Reducing the minimum sentencing or decriminalization of low-level drug offenses could reduce the federal prison population by 50% and state/local prison populations by 15% [26, 27]. Formerly incarcerated individuals face many obstacles when re-entering into society, including disenfranchisement and barriers to employment [27]. Policies promoting employment opportunities for formerly incarcerated individuals could diminish economic inequities resulting from mass incarceration [27].

In addition to the impact on school engagement that having a parent incarcerated has, economic well-being was an important factor in predicting school engagement. Addressing income inequality through policies encouraging economic well-being could have a substantial influence on school engagement. In recent decades, wages for low-income individuals have remained stagnant while higher-income groups have seen massive gains [27]. Minimum wage increase has garnered significant support within the US [34]. Furthermore, expanding the Supplemental Nutrition Assistance Program (SNAP) could decrease food insecurity and increase economic activity [27]. The policy approach should be multi-faceted and incorporate criminal justice reform, economic stimulus, and social programs to minimize the influences of ACEs on school engagement.

### Strengths and limitations

The complex survey design of NSCH allowed for our study to be nationally representative of the US. Additionally, the current study’s innovative use of dominance analysis to understand the relative importance of ACEs in predicting school engagement yielded insights that will guide future research and policy interventions. Despite the noted strengths of the current study, we did have certain limitations. The NSCH made changes to survey questions included in our study from earlier iterations. This restricted our analysis to 2018/2019 data. Since then, the COVID-19 pandemic significantly changed the education landscape in the U.S., resulting in worse school engagement compared to before the onset of the pandemic [35]. Further, the prevalence of ACEs increased during the pandemic pointing towards a compounding impact on worse school engagement and health disparities [36]. While this study did not include data from COVID-19, the effect of the pandemic cannot be understated on ACEs and school engagement; future studies are needed to examine these impacts. Another limitation of the study includes recall bias as the data collection method for the NSCH relies on parental/guardian reported data. Additionally, future studies may investigate additional covariates that may have an influence on school engagement including parental support, number of children in the household, parental immigration status, and primary caregiver physical health.

Furthermore, dominance analysis is a relatively new statistical approach in the field of public health and has its own set of restrictions. Dominance analysis was initially built to be used for ordinary least squares (OLS) multiple linear regression, wherein R^2^ statistics can be used to measure the percent of variance accounted for by a statistical model [37–39]. Consequently, dominance analyses of OLS models can be interpreted in terms of explained variance [38]. However, the nature of our outcome variables required the use of logistic regression. While dominance analysis has been successfully adapted for use with logistic regression, the nature of pseudo-R^2^ statistics results in an inability for us to report the percentage of variance accounted for by each ACE [20].

## Conclusion

Despite the limitations, the results provide unique and actionable insights. Most notably, this study points toward the large influence out-of-school factors play on school engagement. Parental incarceration and economic hardship are issues that can be addressed and mitigated through policy interventions. With limited funds available for education and public health interventions, it is crucial that these two ACEs are tackled through significant policy changes. However, it is important to note that any ACEs can result in worse school engagement and health outcomes, which should be considered when developing interventions. Based on the results from this study, in the context of the study population, a multi-faceted policy approach that reduces the incarcerated population, encourages economic well-being, and emphasizes early-childhood education has the potential to significantly reduce the inequities our society is facing [27].

## Supplementary Information

**Additional file 1.** Logistic regression assumption testing: multicollinearity of the explanatory and covariables form model 1 (outcome: repeated grades). Variance Inflation Factor (VIF) determines the correlation between the independent variables. A score close to 1 indicates that there is little correlation while a score close to 10 indicates a large correlation. This file includes a table that shows the test of the logistic regression assumption of multicollinearity.

**Additional file 2.** Unadjusted bivariate analyses for three model outcomes (repeated grades, does all required homework, and cares about doing well in school) by demographic, ACE, and other covariables for the National Survey of Children’s Health 2018-2019. This file contains descriptive analyses of the explanatory and covariate variables for the three model outcomes.

## Data Availability

The datasets analysed in this study are publicly available through the Health Resources and Services Association (HRSA) using this link https://mchb.hrsa.gov/data/national-surveys.

## Abbreviations

ACEs: Adverse Childhood Experiences
CDC: Centers for Disease Control and Prevention
NSCH: National Survey of Children’s Health
SHCN: Special Health Care Needs
SES: Socio-Economic Status
SNAP: Supplemental Nutrition Assistance Program
