# Supplementary material for: Potential effects of adverse childhood experiences on school engagement in youth: a dominance analysis
Source: BMC Public Health. 2022 Nov 16;22:2096. doi: 10.1186/s12889-022-14524-8 (PMC9668388; doi:10.1186/s12889-022-14524-8)
Supplement: Supplementary file 1 — Additional file 1. Logistic regression assumption testing: multicollinearity of the explanatory and covariables form model 1 (outcome: repeated grades). Variance Inflation Factor (VIF) determines the correlation between the independent variables. A score close to 1 indicates that there is little correlation while a score close to 10 indicates a large correlation. This file includes a table that shows the test of the logistic regression assumption of multicollinearity. [file 12889_2022_14524_MOESM1_ESM.pdf]

Potential Effects of Adverse Childhood Experiences on School Engagement in Youth: A Dominance Analysis

Additional File 1: Logistic regression assumption testing: multicollinearity of the explanatory and covariables form model 1 (outcome: repeated grades). Variance Inflation Factor (VIF) determines the correlation between the independent variables. A score close to 1 indicates that there is little correlation while a score close to 10 indicates a large correlation.

Variable	VIF	Tolerance
Sex	1.00	0.998
Age	1.08	0.927
Race/Ethnicity	1.03	0.966
Minimum Parental Education	1.05	0.949
Health Issue	1.06	0.946
Hard to cover basics like food or housing	1.10	0.906
Treated unfairly because of race	1.07	0.933
Parent or guardian divorce	1.28	0.784
Parent or guardian death	1.04	0.965
Parent or guardian in jail	1.37	0.732
Adults slap, hit, kick, punch others	1.40	0.717
Victim of violence	1.24	0.806
Lived with mentally ill	1.26	0.796
Lived with person with alcohol/drug problem	1.46	0.683
