# Supplementary material for: Potential effects of adverse childhood experiences on school engagement in youth: a dominance analysis
Source: BMC Public Health. 2022 Nov 16;22:2096. doi: 10.1186/s12889-022-14524-8 (PMC9668388; doi:10.1186/s12889-022-14524-8)
Supplement: Supplementary file 2 — Additional file 2. Unadjusted bivariate analyses for three model outcomes (repeated grades, does all required homework, and cares about doing well in school) by demographic, ACE, and other covariables for the National Survey of Children’s Health 2018-2019. This file contains descriptive analyses of the explanatory and covariate variables for the three model outcomes. [file 12889_2022_14524_MOESM2_ESM.pdf]

Potential Effects of Adverse Childhood Experiences on School Engagement in Youth: A Dominance Analysis

Additional File 2: Unadjusted bivariate analyses for three model outcomes (repeated grades, does all required homework, and cares about doing well in school) by demographic, ACE, and other covariables for the National Survey of Children's Health 2018-2019.

		Outcome 1: Repeated Grade			Outcome 2: Does All Required Homework			Outcome 3: Cares About Doing Well in School		
Variables	Total by Category n=39,347 * % (95% CI)**	Grade Repeated by Category n=2,201* 6.3% (95% CI)**	Grade Not Repeated by Category n= 37,146* 93.7% (95% CI)**	p-value	Does All Homework by Category n=34,828 * 88.7% (95% CI)**	Does Not Do All Homework by Category n=4,519 * 11.3% (95% CI)**	p-value	Cares About School by Category n= 34,851* 89.1% (95% CI)**	Does Not Care About School by Category n= 4,496 * 10.9% (95% CI)**	p-value
Sex										
Male	50.9 (49.8, 52.0)	64.2 (59.5, 68.7)	50.0 (48.9, 51.2)	<0.0001	48.9 (47.8, 50.1)	66.7 (63.8, 69.6)	<0.0001	48.8 (47.7, 50.0)	68.2 (65.3, 70.9)	<0.0001
Female	49.1 (48.0, 50.2)	35.8 (31.3, 40.5)	50.0 (48.9, 51.1)		51.1 (49.9, 52.3)	33.3 (30.4, 36.2)		51.2 (50.0, 52.4)	31.8 (29.1, 34.7)	
Age										
6-9	32.5 (31.5, 33.6)	19.1 (16.0, 22.7)	33.4 (32.4, 34.5)	<0.0001	33.9 (32.8, 35.0)	21.9 (19.3, 24.8)	<0.0001	33.2 (32.1, 34.3)	26.8 (24.1, 29.6)	<0.0001
10-13	34.2 (33.2, 35.2)	34.9 (30.6, 39.5)	34.1 (33.1, 35.2)		34.1 (33.0, 35.2)	34.6 (31.8, 37.5)		34.2 (33.1, 35.3)	34.4 (31.6, 37.2)	

14-17	33.3 (32.3, 34.3)	46.1 (41.3, 50.9)	32.5 (31.5, 33.5)		32.0 (31.0, 33.1)	43.5 (40.5, 46.5)		32.6 (31.6, 33.7)	38.9 (36.0, 41.8)	
Race/Ethnicity										
White	51.4 (50.3, 52.5)	43.1 (38.7, 47.5)	51.9 (50.8, 53.0)	0.0001	51.7 (50.6, 52.9)	48.6 (45.7, 51.6)	<0.0001	51.4 (50.2, 52.5)	51.7 (48.6, 54.7)	0.0036
Black	12.8 (12.1, 13.6)	17.9 (14.8, 21.4)	12.5 (11.7, 13.3)		12.1 (11.3, 12.9)	18.9 (16.4, 21.6)		12.5 (11.7, 13.3)	15.5 (13.3, 18.0)	
Hispanic	25.2 (24.1, 26.5)	30.5 (25.3, 36.1)	24.9 (23.7, 26.1)		25.4 (24.1, 26.7)	24.1 (21.0, 27.5)		25.5 (24.2, 26.8)	23.3 (20.2, 26.7)	
Other	5.1 (4.7, 5.5)	4.8 (3.4, 6.8)	5.1 (4.7, 5.5)		5.0 (4.6, 5.4)	5.6 (4.5, 6.8)		5.0 (4.5, 5.4)	6.0 (4.9, 7.2)	
>=2 or more races	5.5 (5.1, 5.9)	3.8 (2.6, 5.4)	5.6 (5.2, 6.1)		5.8 (5.4, 6.3)	2.8 (3.1, 3.7)		5.7 (5.3, 6.2)	3.6 (2.6, 4.9)	
Minimum Parental Education										
Less than high school	17.9 (16.7, 19.1)	32.4 (27.4, 37.8)	16.9 (15.8, 18.1)	<0.0001	17.4 (16.2, 18.7)	21.8 (18.8, 25.1)	<0.0001	17.5 (16.3, 18.8)	20.7 (17.8, 24.0)	<0.0001
HS/GED/Vocational	29.2 (28.2, 30.2)	36.3 (32.0, 40.8)	28.7 (27.7, 29.7)		28.6 (27.6, 29.7)	33.9 (31.1, 36.7)		28.4 (27.4, 29.5)	35.6 (32.8, 38.5)	
Some college	22.6 (21.8, 23.4)	17.6 (15.2, 20.3)	22.9 (22.1, 23.7)		22.5 (21.6, 23.3)	23.6 (21.4, 25.9)		22.4 (21.6, 23.3)	24.0 (21.8, 26.3)	
Bachelor	21.2 (20.5, 22.0)	10.5 (8.5, 12.9)	21.9 (21.2, 22.7)		22.0 (21.2, 22.8)	15.3 (13.4, 17.4)		22.0 (21.2, 22.8)	14.7 (13.1, 16.5)	
Masters or greater	9.1 (8.6, 9.7)	3.2 (2.4, 4.4)	9.5 (9.0, 10.1)		9.6 (9.1, 10.2)	5.5 (4.4, 6.9)		9.6 (9.1, 10.2)	5.1 (4.2, 6.2)	

Health Issue									
None	71.6 (70.7, 72.5)	52.3 (47.6, 57.0)	72.9 (72.0, 73.8)		75.5 (74.6, 76.4)	41.0 (37.9, 44.1)		75.5 (74.6, 76.4)	40.1 (37.2, 43.1)	
Asthma, no ADHD	7.9 (7.3, 8.5)	6.4 (5.0, 8.1)	8.0 (7.4, 8.6)		7.9 (7.3, 8.5)	7.6 (6.3, 9.2)		7.9 (7.3, 8.5)	7.6 (6.2, 9.3)	
ADHD, no asthma	8.9 (8.4, 9.4)	21.1 (17.8, 25.1)	8.0 (7.6, 8.6)	<0.0001	6.3 (5.9, 6.8)	28.7 (26.1, 31.5)	<0.0001	6.4 (5.9, 1.6)	29.3 (26.6, 32.1)	<0.0001
Both Asthma, ADHD	1.8 (1.5, 2.1)	4.8 (3.4, 6.7)	1.6 (1.4, 1.9)		1.2 (1.0, 1.4)	6.5 (5.2, 8.1)		1.3 (1.1, 1.7)	5.8 (4.5, 7.3)	
Neither, other SHCN	9.9 (9.3, 10.5)	15.3 (12.1, 19.2)	9.5 (9.0, 10.1)		9.1 (8.5, 9.7)	16.2 (14.4, 18.2)		9.0 (8.4, 9.6)	17.3 (15.2, 19.6)	
Adverse Childhood Experiences										
Hard to cover basics like food or housing										
No	84.4 (83.5, 85.2)	74.1 (69.8, 77.9)	85.0 (84.2, 85.9)	<0.0001	86.3 (85.5, 87.2)	68.9 (66.0, 71.6)	<0.0001	86.1 (85.2, 86.9)	70.3 (67.5, 73.0)	<0.0001
Yes	15.6 (14.8, 16.5)	25.9 (22.1, 30.2)	15.0 (14.2, 15.8)		13.7 (12.9, 14.5)	31.1 (28.4, 34.0)		13.9 (13.1, 14.8)	29.7 (27.0, 32.5)	
Treated unfairly because of race										
No	94.2 (93.6, 94.7)	89.1 (85.5, 91.9)	94.5 (94.0, 95.1)	<0.0001	94.9 (94.3, 95.5)	88.6 (86.2, 90.7)	<0.0001	94.6 (94.0, 95.2)	90.8 (88.8, 95.6)	<0.0001
Yes	5.8 (5.3, 6.4)	10.9 (8.1, 14.5)	5.5 (4.9, 6.0)		5.1 (4.6, 5.7)	11.4 (9.3, 13.8)		5.4 (4.8, 6.0)	9.2 (7.5, 11.2)	
Parent or guardian divorce										

No	71.2 (70.2, 72.2)	59.7 (55.3, 64.1)	72.0 (71.0, 73.0)	<0.0001	73.3 (72.3, 74.3)	55.0 (52.0, 58.0)	<0.0001	73.1 (72.1, 74.1)	55.7 (52.8, 58.7)	<0.0001
Yes	28.8 (27.8, 29.8)	40.3 (36.0, 44.7)	28.0 (27.0, 29.0)		26.7 (25.7, 27.7)	45.0 (42.1, 48.0)		26.9 (25.9, 27.9)	44.3 (41.3, 47.3)	
Parent or guardian death										
No	96.1 (95.7, 96.5)	91.5 (87.9, 94.0)	96.5 (96.1, 96.8)	<0.0001	96.4 (96.0, 96.8)	94.0 (92.6, 95.2)	<0.0001	96.4 (96.0, 96.8)	94.2 (92.8, 95.3)	0.0001
Yes	3.9 (3.5, 4.3)	8.6 (6.0, 12.1)	3.6 (3.2, 3.9)		3.6 (3.2, 4.0)	6.0 (4.8, 7.4)		3.6 (3.2, 4.0)	5.8 (4.7, 7.2)	
Parent or guardian time in jail										
No	91.3 (90.7, 91.9)	80.7 (77.1, 83.8)	92.0 (91.5, 92.6)	<0.0001	92.6 (92.0, 93.1)	81.5 (79.0, 93.7)	<0.0001	92.4 (91.8, 92.9)	82.8 (80.4, 85.0)	<0.0001
Yes	8.7 (8.1, 9.3)	19.3 (16.2, 22.9)	8.0 (7.4, 8.6)		7.4 (6.9, 8.0)	18.5 (16.3, 21.0)		7.6 (7.1, 8.2)	17.2 (15.0, 19.6)	
Adults slap, hit, kick, punch others										
No	93.2 (92.7, 93.8)	85.3 (81.8, 88.2)	93.8 (93.2, 94.3)	<0.0001	94.3 (93.7, 94.8)	85.0 (82.6, 87.1)	<0.0001	94.1 (93.5, 94.6)	86.3 (84.1, 88.2)	<0.0001
Yes	6.8 (6.2, 7.3)	14.7 (11.8, 18.2)	6.2 (5.7, 6.8)		5.7 (5.2, 6.3)	15.0 (12.9, 17.4)		5.9 (5.4, 6.5)	13.7 (11.8, 15.9)	
Victim of Violence										
No	94.8 (94.3, 95.3)	89.6 (86.9, 91.8)	95.1 (94.6, 95.6)	<0.0001	95.8 (95.3, 96.3)	86.7 (84.4, 88.6)	<0.0001	95.7 (95.1, 96.1)	87.6 (85.4, 89.6)	<0.0001

Yes	5.2 (4.7, 5.7)	10.4 (8.2, 13.1)	4.9 (4.4, 5.4)		4.2 (3.7, 4.7)	13.3 (11.4, 15.6)		4.4 (3.9, 4.9)	12.4 (10.4, 14.7)	
Lived with mentally ill										
No	90.4 (89.7, 91.0)	82.0 (78.0, 85.5)	90.9 (90.3, 91.5)	<0.0001	91.8 (91.1, 92.4)	79.4 (77.1, 81.5)	<0.0001	91.6 (90.9, 92.2)	80.4 (78.1, 82.6)	<0.0001
Yes	9.6 (9.0, 10.3)	18.0 (14.5, 22.0)	9.1 (8.5, 9.7)		8.2 (7.6, 8.9)	20.6 (18.5, 22.9)		8.4 (7.8, 9.1)	19.6 (17.4, 21.9)	
Lived with person with alcohol/drug problem										
No	89.8 (89.1, 90.4)	82.5 (78.2, 86.1)	90.2 (89.6, 90.9)	<0.0001	90.9 (90.2, 91.5)	80.9 (78.8, 83.0)	<0.0001	90.9 (90.2, 91.5)	80.7 (78.3, 82.8)	<0.0001
Yes	10.2 (9.6, 10.9)	17.5 (13.9, 21.8)	9.8 (9.1, 10.4)		9.1 (8.5, 9.8)	19.1 (17.1, 21.3)		9.1 (8.5, 9.8)	19.3 (17.2, 21.7)	

*Unweighted NSCH population size

**Weighted percentage and 95% confidence interval
